# The effects of weight categories on the time-motion analysis of female high-level judo athletes between the 2016 and 2020 Olympic cycles

**DOI:** 10.3389/fpsyg.2022.1012517

**Published:** 2022-12-05

**Authors:** Lindsei Brabec Mota Barreto, Bianca Miarka, Roberto Jerônimo dos Santos Silva, Nicola Luigi Bragazzi, Maamer Slimani, Hela Znazen, Dani Alexis Sorbazo Soto, Esteban Ariel Aedo-Muñoz, Ciro Jose Brito

**Affiliations:** ^1^Department of Physical Education, Federal University of Juiz de Fora, Campus Governador Valadares, Governador Valadares, Brazil; ^2^Department of Physical Education, Laboratory of Psychophysiology and Performance in Sports & Bouts, Postgraduate Program in Physical Education, School of Physical Education and Sport, Federal University of Rio de Janeiro, Rio de Janeiro, Brazil; ^3^Department of Physical Education, Federal University of Sergipe, Aracaju, Brazil; ^4^Laboratory for Industrial and Applied Mathematics (LIAM), Department of Mathematics and Statistics, York University, Toronto, ON, Canada; ^5^Department of Neuroscience, Rehabilitation, Ophthalmology, Genetics, Child and Maternal Health, Faculty of Medical and Pharmaceutical Sciences, University of Genoa, Genoa, Italy; ^6^Department of Physical Education and Sport, College of Education, Taif University, Taif, Saudi Arabia; ^7^Escuela de Kinesiología, Facultad de Salud, Magister en Ciencias la Actividad Física y Deportes Aplicadas al Entrenamiento Rehabilitación y Reintegro Deportivo, Universidad Santo Tomás, Puerto Montt, Chile; ^8^Universidad Metropolitana de Ciencias de la Educación, Santiago, Chile

**Keywords:** martial arts, match analysis, time and motion studies, task performance and analysis, gender

## Abstract

This study compared the time of female judo combat phases in international competitions between two Olympic cycles (2016; 2020) according to weight divisions (48 kg = 132; 52 kg = 72; 57 kg = 109; 63 kg = 96; 70 kg = 69; 78 kg = 106; >78 kg = 82; total = 666 combats/cycle). The behaviors of 1,332 high-level female judo combats were randomly observed over two Olympic cycles (2016 = 666; 2020 = 666) from the top 20 athletes in the world ranking by weight division. We performed time-motion analysis according to the combat phase and sequential judo actions (approach, gripping, attack, defense, groundwork, pause, and effort: pause ratio) considering the moment when the combat ended (Regular time = RT; Golden score = GS). The weight division groups were compared between Olympic cycles (2016; 2020), and *p* < 0.05 was defined as significant. The main results showed that 2020 athletes spent less time in the gripping (*p* = 0.005), attack (*p* < 0.001), defense (*p* < 0.001), groundwork (*p<*0.001) and pause (*p* = 0.002) phases than 2016 athletes. However, compared by the end-of-combat, 2020 female athletes spent less time in all combat phases for RT combats (*p* < 0.001), and more time in the approach phase for GS combats (*p* < 0.05) than in 2016. The 2016 weight divisions showed a higher diversity in the effort: pause ratio (2.5:1–3.4:1), whereas the 2020 weight divisions had values closer to each other (2.8,1–3:1). Analyzing each weight division separately and by the end-of-combat, the main results showed that (*p* < 0.05): 48, 63, 70, and 78 kg reduced the time in almost every phase of RT combat (except for: 63 kg = gripping and attack; 70 kg = approach and groundwork; 78 kg = approach); 48 and 57 kg increased the groundwork time in GS combats whereas 78 kg decreased; 52 kg and 78 kg increased the GS approach time. The temporal behavior of the combats changed between the Olympic cycles with different rules. These data must be considered to understand the characteristics of each group and to prescribe specialized training in female judo.

## Introduction

Notational analysis allows us to understand how competitive actions are developed in a combat sport (Miarka et al., 2022). This technical analysis allows the most effective actions applied by fighters to be identified (Miarka et al., 2016; Brito et al., 2017), differentiating the time and frequency dedicated to each action that can lead the athlete to achieve victory (Barreto et al., 2021). When the coach has access to accurate information about the weight division in which his athlete competes, an efficient and contextualized training plan can be prepared so that the training load is adequate to the athlete’s competitive demand (Miarka et al., 2016, 2020; Brito et al., 2017, 2020). To create more specific and, consequently, more efficient judo training, each temporal phase of combat should be known (Segedi et al., 2014; Sterkowicz-Przybycien et al., 2017); these phases can be classified into approach, gripping, attack, defense, groundwork and pause (Miarka et al., 2011, 2014). The technical-tactical behavior of athletes has been described by studies that temporally characterized the combat phases to identify behaviors that can predict the best sports performance (Kajmovic and Radjo, 2014; Miarka et al., 2014, 2016).

However, continuous rule changes made by the International Judo Federation might have caused changes in the configuration of these temporal phases. In 2015, female judo combat time was reduced from 5 to 4 min, and in 2017–2018, the Yuko score was abolished, the number of penalties (*Shido*) decreased from 4 to 3 (International Judo Federation, 2017a), and the winner was no longer determined at the end of the combat or by the Golden Score (International Judo Federation, 2017b). Due to these changes in the rules, some researchers have attempted to measure the effect of these changes on the behavior of athletes during judo combat (Calmet et al., 2017a,b; Ceylan and Balci, 2017; Barreto et al., 2021, 2022a). To this end, Ceylan and Balci (2017) compared the 2016 and 2017 Paris Grand Slam tournaments; they observed that changing the rules increased the frequency of *Wazari* (half a point) in male and female judokas and decreased the frequency of *Shido* in male judokas. Moreover, they verified that the current rules increase the frequency of combats decided in the GS for male athletes.

Between the 2016 (Rio de Janeiro) and 2021 (Tokyo) Olympic games, rule changes were implemented in 2017, 2018 and 2020 (Barreto et al., 2022b). However, to the best of our knowledge, no technical-tactical analysis studies were carried out in female judo that compared whether these changes in rules resulted in differences in the behavior of athletes. Given the importance of this knowledge for the development of more up-to-date training, new studies analyzing the technical-tactical behavior of fighters in the current rules are interesting (Barreto et al., 2021, 2022a), especially in female judo competition. In this context, the objective of this study was to analyze the temporal phases of international female judo combat in two Olympic cycles (2016 vs. 2020) with different rules by weight division. The data from this study can be useful for judo coaches to plan training considering the temporal demand of each judo combat phase by weight division. We hypothesized that the rule changes affect the combat time dedicated to each combat phase.

## Materials and methods

### Sample

The present study analyzed 1,332 combat videos of female judo from two Olympic cycles (2016 vs. 2020) distributed in identical numbers by weight division (48 kg = 132; 52 kg = 72; 57 kg = 109; 63 kg = 96; 70 kg = 69; 78 kg = 106; ˃78 kg = 82; total = 666 combats/cycle). The athletes analyzed were among the top 20 of each weight division in the World Ranking (ranking of May 30, 2016; March 16, 2020). The 2016 cycle combats were collected after the 2015 rule change (years 2015 and 2016), so the regular combat time was 4 min. The 2020 cycle combats took place before the interruption of events due to the COVID-19 pandemic (years 2019 and 2020, until January 25, 2020). In the first 2 years of an Olympic cycle in which there is a rule change, athletes need a phase of adaptation to this new context. Therefore, in the 2016 and 2020 cycles, we collected combats from the last 2 years (considering that athletes would already be familiar with the new rules).

The combat videos had a minimum quality of 480/60 pixels, a panoramic view of the entire competition area and were available for public access on the virtual YouTube channel of the International Judo Federation and the Olympic Committee (available in https://www.youtube.com/channel/UCTl3QQTvqHFjurroKxexy2Q; https://www.youtube.com/c/judo/videos); therefore, obtaining informed consent from the athletes was not necessary. The number of combats analyzed per Olympic cycle was determined from the collection of all combats found from the 2016 cycle from the top 20 athletes of each weight division in the World Ranking, since for this period there were fewer videos available on the internet compared to the 2020 cycle. Thus, the combats were analyzed in identical amounts per Olympic cycle, according to sex and weight division.

The combats were from the following international judo competitions: 26 Grand Prix (Almaty 2016; Antalaya 2019; Budapest 2015, 2016, 2019; Dusseldorf 2015, 2016; Havana 2016; Hohhot 2019; Jeju 2015; Marrakech 2019; Montreal 2019; Qingdao 2015, 2016; Samsun 2015, 2016; Tashkent 2016, 2019; Tbilisi 2015, 2016, 2019; Tel Aviv 2019, 2020; Ulaanbaatar 2016; Zagreb 2016, 2019); 11 Grand Slam (Abu Dhabi 2015, 2016, 2019; Baku 2015, 2016, 2019; Paris 2016; Tokyo 2015, 2016; Tyumen 2015, 2016); 2 World Championship (Astana 2015; Tokyo 2019); and the Rio 2016 Olympic Games. The distribution of combats by competition between the 2016 and 2020 cycles was not the same (Grand Prix: 2016 cycle = 16, 2020 cycle = 10; Grand Slam: 2016 cycle = 9, 2020 cycle = 2, Olympic Games: only in the 2016 cycle; World Championship: 1 for both cycles). This distribution is due to the existence of combats available on the internet (in full) of the 20 best ranked athletes in each weight division, in each cycle. Data from the 2020 Olympics was not collected because it was postponed due to the covid-19 pandemic, it taking place in 2021 (outside the period of the last 2 years of an Olympic cycle, inclusion criteria for combat videos).

### Procedures

We used a validated analysis protocol for judo (Miarka et al., 2011, 2015), which divided the judo combats into approach (combat beginning by the *hajime* command until the grip on the opponent’s uniform—*judogi* grip), grip (characterized by the minimum permanence of 1 s of grip on the *judogi*), attack (performing techniques falling), groundwork (movements with at least three body parts on the ground) and pause phases (interruption of the combat by the referee; Miarka et al., 2011, 2014, 2015).

To analyze the videos, we used the Frami^®^ software and the media player VLC 3.0.4 to make the video compatible with this software (Miarka et al., 2011, 2015). In this study, the time spent in each combat phase was established by weight division, by Olympic cycle (2016 vs. 2020), and by moment of combat end [regular time (RT) or golden score (GS)]. We also calculated the effort: pause ratio by dividing the sum of time spent in action phases by the pause time.

The videos were analyzed by a judo expert (˃ 25 years of Judo, black 2nd Dan degree, national competitive experience) who was trained to use the Frami^®^ and the analysis protocol for 12 h. The use of the judo analysis protocol was demonstrated to be objective when performed by experts with a minimum degree of brown (1st Kyu) and at least 7 h of training in Frami (Miarka et al., 2011; Ando et al., 2016). In fact, the reliability of the analysis was verified (20 judo combats reanalyzed 1 week later), and agreement was “excellent” for all combat phases (intraclass correlation coefficient = 0.95; 0.99; confidence interval = 0.88; 1).

### Statistical analysis

For the statistical analysis, we used the SPSS software (version 20.0; SPSS, Inc., Chicago, IL, United States) with a significance level of *p* ≤ 0.05. The reliability of the use of the video analysis protocol was calculated with the intraclass correlation coefficient test and confidence interval, as the data are quantitative. In the descriptive analysis of the temporal data of each judo combat phase (in seconds), we used the mean, standard deviation and interval. Kolmogorov–Smirnov test was used to verify the normality of the data. According to the normality check for each variable, we use Student’s t test for independent samples or Mann–Whitney *U* test to analyze the data by the Olympic cycle and by weight division. The effect size was calculated and classified considering: ≤ 0.1 = small effect; 0.11–0.3 = mean effect; 0.31–0.5 = great effect.

## Results

Table 1 shows the time of each phase of female judo combat by Olympic cycle (2016 vs. 2020) and by weight division. Athletes from 2020 cycle spent less time in the gripping (*U* = 202229.5; *p* = 0.005; *r* = −0.008), attack (*U* = 191004.5; *p* < 0.001; *r* = −0.12), defense (*U* = 192011.5; *p* < 0.001; *r* = −0.12), groundwork (*U* = 195862.5; *p* < 0.001; *r* = −0.08) and pause (*U* = 200080.5; *p* = 0.002; *r* = −0.08) phases than athletes from 2016 cycle. In the analysis by weight division, whereas the effort/pause ratio varied in the 2016 cycle (2.5:1–3.4:1), since the lowest effort/pause ratio was at 63 kg (2.5:1) and the highest was at 52 kg (3.4:1), these values were similar between divisions in the 2020 cycle (2.8:1–3:1).

**Table 1:** Time of combat phases of female judo in the 2016 and 2020 Olympic cycles (*n* = 1,332).

Weight divisions (Combats per cycle)	Combat phases time (seconds) (mean ± standard deviation/interval)							Effort:pause ratio
	Approach	Gripping	Attack	Defense	Groundwork	Pause		
2016 cycle	All categories (*n* = 666)	69 ± 38.2/250	76.2 ± 42.9/289^#^	4.7 ± 4.2/29^*^	4.6 ± 4.4/32^*^	49.9 ± 31/207^*^	73.3 ± 54.4/345^$^	2.7:1
	48 kg (*n* = 132)	86.3 ± 39.1/227	63.4 ± 30.6/143	6.3 ± 5.2/29^&^	6 ± 5.5/32^ω^	64.1 ± 25.7/137	83.3 ± 47.5/215^§^	2.7:1
	52 kg (*n* = 72)	68.8 ± 35.2/139	72.2 ± 38.7/156^ж^	4.7 ± 3.9/21	4.3 ± 3.7/17	57.6 ± 35/159	61.1 ± 38.6/178	3.4:1
	57 kg (*n* = 109)	70.8 ± 43/250	80.6 ± 48/289	4.3 ± 3.9/19	4.3 ± 3.9/17	43.4 ± 32/151^α^	65.6 ± 46/188	3.1:1
	63 kg (*n* = 96)	66.9 ± 32.9/137	67.1 ± 38.4/169	4.8 ± 4.1/19	5.1 ± 4.5/22^₡^	51.9 ± 31.8/138	78.8 ± 55/278	2.5:1
	70 kg (*n* = 69)	67.1 ± 42.7/182^μ^	74 ± 40.7/189^◘^	4.4 ± 4.1/21	4.5 ± 4.3/23	46.4 ± 30/122	72.6 ± 66/339	2.7:1
	78 kg (*n* = 106)	57.9 ± 27.4/116	91.3 ± 40.8/164	4.3 ± 4.2/20	4.3 ± 4.3/23	44.4 ± 33/122^*^	76.4 ± 49/224	2.6:1
	˃78 kg (*n* = 82)	57.6 ± 37.2/215^∞^	87.4 ± 55.9/251^ʒ^	3 ± 2.4/10	2.7 ± 2.6/12	36.5 ± 18.3/82^*^	68.6 ± 75.8/345	2.7:1
2020 cycle	All categories (*n* = 666)	72.8 ± 51.4/309	72.2 ± 50.2/321^#^	3.6 ± 3.3/23^*^	3.6 ± 3.7/25^*^	45 ± 34.1/186^*^	69.3 ± 60.9/314^$^	2.8:1
	48 kg (*n* = 132)	86.8 ± 55.3/259	70.2 ± 44.4/201	4.4 ± 3.9/17^&^	4.5 ± 4.2/22^ω^	59.7 ± 38.6/183	81.3 ± 70.9/270^§^	2.8:1
	52 kg (*n* = 72)	84.4 ± 65.1/283	62 ± 46.6/252^ж^	3.7 ± 2.9/17	3.8 ± 3.5/16	51.3 ± 33.1/143	69.7 ± 55.7/246	2.9: 1
	57 kg (*n* = 109)	83.4 ± 61/309	78.2 ± 55.5/223	3.8 ± 3.8/23	3.8 ± 4.1/25	55.8 ± 35.2/174^α^	78.4 ± 67.9/314	2.9: 1
	63 kg (*n* = 96)	71.4 ± 46.4/247	70.9 ± 46.9/243	4.2 ± 3.8/19	3.8 ± 4.1/22^₡^	45.3 ± 27.2/126	71.1 ± 60.9/307	2.8: 1
	70 kg (*n* = 69)	57.5 ± 35.8/155^μ^	55.9 ± 45.7/205^◘^	3.1 ± 2.7/13	3.2 ± 3.7/22	43.1 ± 37.4/183	54.9 ± 48.4/186	3: 1
	78 kg (*n* = 106)	66.7 ± 42.6/217	89.2 ± 60.9/317	3.5 ± 2.7/12	3.4 ± 2.9/14	27.7 ± 23.6/108^*^	67.4 ± 57.2/258	2.8: 1
	˃ 78 kg (*n* = 82)	48.2 ± 25.7/130^∞^	69.4 ± 40/154^ʒ^	2.1 ± 2.2/10	2.4 ± 2.5/11	25.3 ± 20.8/119^*^	49.8 ± 44.1/204	3: 1

Significant difference between 2016 vs. 2020 cycle: ^*^p<0.001; ^&^*p* = 0.001; ^$^*p* = 0.002; ^μ^*p* = 0.003; ^#^*p* = 0.005; ^α^*p* = 0.007; ^ʒ^*p* = 0.008; ^ω^*p* = 0.014; ^∞^*p* = 0.019; ^₡^*p* = 0.027; ^◘^*p* = 0.028; ^§^*p* = 0.035; ^ж^*p* = 0.041.

In addition, the following situations significantly differed between the Olympic cycles, and 2020 cycle athletes: from 48 kg spent less time for the attack (*U* = 6,608; *p* = 0.001; *r* = −0.21), defense (*U* = 7,196; *p* = 0.014; *r* = −0.15) and pause (*U* = 7404.5; *p* = 0.035; *r* = −0.13) phases; from 52 kg spent less time in the gripping phase (*U* = 2080; *p* = 0.041; *r* = −0.17); from 57 kg spent more time in the groundwork phase (*t*_(216)_ = −2.733; *p* = 0.007; *r* = −0.18); from 63 kg spent less time in the defense phase (*U* = 3,762; *p* = 0.027; *r* = −0.16); from 70 kg spent less time in the gripping (*U* = 1,692; *p* = 0.003; *r* = −0.25) and attack (*t*_(136)_ = 2.219; *p* = 0.028; *r* = 0.19) phases; from 78 kg spent less time in the groundwork phase (*U* = 3813.5; *p* < 0.001; *r* = −0.28); from ˃ 78 kg spent less time in the gripping (*t*_(162)_ = 2.365; *p* = 0.019; *r* = 0.18), attack (*U* = 2,558; *p* = 0.008; *r* = 0.21) and groundwork phase (*t*_(162)_ = 3.681; *p* < 0.001; r = 0.28; Table 1).

Table 2 shows the time phases, by weight division, from combats finished in RT (2016 cycle = 91%; 2020 cycle = 79.6% of the combats) or GS (2016 cycle = 9%; 2020 cycle = 20.4% of the combats) in each Olympic cycle. In combat trials that continued until RT, 2020 athletes spent less time in all combat phases than 2016 athletes (approach: *U* = 134,434; *r* = −0.13; gripping: *U* = 124894.5; *r* = −0.18; attack: *U* = 126,715; *r* = −0.17; defense: *U* = 125168.5; *r* = −0.18; groundwork: *U* = 126,674; *r* = −0.17; pause: *U* = 122,365; *r* = −0.19; *p* < 0.001 for all). In combats ended in the GS, the 2020 athletes spent more time in the approach phase (*t*_(194)_ = −2.233; *p* = 0.027; *r* = 0.16) than 2016 athletes.

**Table 2:** Time of combat phases of female judo, separating the combats by ending moment, between the 2016 and 2020 Olympic cycles (*n* = 1,332).

Weight divisions (Combats per cycle)		Combat phases time (seconds) (mean ± standard deviation)												Effort: pause ratio	
		Approach		Gripping		Attack		Defense		Groundwork		Pause			
		RT	GS	RT	GS	RT	GS	RT	GS	RT	GS	RT	GS	RT	GS
2016 cycle	All categories (*n* = 666)	63.7 ± 32.2^*^	123.3 ± 49.9^#^	72.2 ± 39.2^*^	116.2 ± 56.5	4.4 ± 4.1^*^	7.6 ± 4.8	4.3 ± 4.2^*^	7.2 ± 4.8	47.4 ± 28.9^*^	75.5 ± 39.2	66.2 ± 48.6^*^	145.3 ± 57.7	2.9:1	2.3/1
	48 kg (*n* = 132)	78.2 ± 149.7^*^	149.7 ± 48.1	62.7 ± 30.9^ʮ^	68.6 ± 28.7^*^	6 ± 5^*^	8.6 ± 6.2	5.7 ± 5.4^*^	8.3 ± 6	62.4 ± 26.2^*^	78.1 ± 16.3^ω^	76.1 ± 44.4^*^	139.7 ± 30.5	2.8: 1	2.2:1
	52 kg (*n* = 72)	61.4 ± 30.3	121.1 ± 19.6^$^	66.3 ± 35.8^β^	114.2 ± 33.1	4.2 ± 3.4^Ξ^	8.4 ± 4.9	3.7 ± 3.1	8.3 ± 5.1	52.5 ± 32.4	93.6 ± 33	53.3 ± 32.3	115.7 ± 35.8	3.5:1	3:1
	57 kg (*n* = 109)	63.2 ± 33.5	119 ± 62.7	70.8 ± 38.4	141.8 ± 57.4	3.8 ± 3.8	7.4 ± 3.8	3.8 ± 3.8	7.1 ± 3.1	40 ± 27.8	64.6 ± 47.1^ж^	53.5 ± 34.2	141 ± 39.1	3.4:1	2.4:1
	63 kg (*n* = 96)	64.8 ± 32^Ω^	104.5 ± 26.6	63.3 ± 35.1	136.6 ± 30.3	4.7 ± 4	8.2 ± 4.4	5 ± 4.4^μ^	7.2 ± 5.9	52.1 ± 31.9^◘^	48.3 ± 32.3	75.9 ± 53.5^β^	132.6 ± 60.2	2.5:1	2.3:1
	70 kg (*n* = 69)	59.5 ± 37.3	124.7 ± 38.1	69 ± 36.6^*^	112.2 ± 52	4 ± 3.7^α^	7.8 ± 5.7	4 ± 3.9^◘^	8.3 ± 5.3	42.8 ± 28.6	74 ± 27.7	57.8 ± 44.2^∞^	185.1 ± 96.1	3.1:1	1.8:1
	78 kg (*n* = 106)	58.1 ± 27.8	51.4 ± 12.8^€^	90.8 ± 41^*^	108.1 ± 32.4	4.3 ± 4.2^$^	3.1 ± 2.7	4.3 ± 4.3^&^	3.4 ± 3.4	41 ± 26.2^*^	159.7 ± 40.5^&^	75.1 ± 48.7^*^	120.2 ± 44.9	2.6:1	2.7:1
	>78 kg (*n* = 82)	53.5 ± 31.6	120.3 ± 61.1	81.6 ± 48.4	176.1 ± 91.1	2.8 ± 2.3^Ł^	6.2 ± 1.9	2.7 ± 2.7	3.2 ± 1.5	35.9 ± 18.5^μ^	46.7 ± 10.5	60.5 ± 67.9	192.9 ± 91.1	2.9:1	1.8:1
2020 cycle	All categories (*n* = 666)	55 ± 30.5^*^	142.5 ± 57.2^#^	57.2 ± 36.4^*^	130.9 ± 54	2.8 ± 2.5^*^	6.7 ± 4.3	2.8 ± 2.7^*^	7.1 ± 4.9	37.4 ± 28.1^*^	74.9 ± 39.1	49.8 ± 42.5^*^	146 ± 61.7	3.1:1	2.5:1
	48 kg (*n* = 132)	61.6 ± 33.3^*^	149 ± 49.3	52.9 ± 31.6^ʮ^	113.1 ± 40.8^*^	3.1 ± 2.8^*^	7.6 ± 4.2	3.2 ± 3.2^*^	7.6 ± 4.8	44.6 ± 26.7^*^	97.2 ± 38.3^L^	50.8 ± 47.1^*^	156.8 ± 63.2	3.3:1	2.4:1
	52 kg (*n* = 72)	58.7 ± 32.1	174.4 ± 72.2^$^	46.6 ± 28.6 ^β^	115.8 ± 57.6	3 ± 2^Ξ^	6.2 ± 4.1	2.6 ± 2.2	8.2 ± 3.9	45.2 ± 32.4	72.5 ± 27.2	47.2 ± 29.6	148.4 ± 54.7	3.3:1	2.5:1
	57 kg (*n* = 109)	62.8 ± 36.1	164.9 ± 71.5	60.2 ± 39.1	149.7 ± 53.6	2.7 ± 2.5	7.9 ± 5	2.9 ± 2.8	7.3 ± 6.1	47.1 ± 28.7	90.1 ± 38.1^ж^	57.1 ± 47.1	162.6 ± 73.1	3.1:1	2.6:1
	63 kg (*n* = 96)	53 ± 26.1 ^Ω^	137 ± 43.8	57.4 ± 36.5	119 ± 49.3	3.4 ± 3	6.9 ± 4.8	2.7 ± 2.9^μ^	7.7 ± 5.3	39.8 ± 24.9^◘^	64.9 ± 26.1	52.5 ± 46.3^β^	137.7 ± 61.2	3:1	2.4:1
	70 kg (*n* = 69)	47 ± 25.2	107.3 ± 37.4	41.4 ± 30.7^*^	124.4 ± 43.4	2.7 ± 2.3^α^	5.1 ± 3.6	2.4 ± 2.1^◘^	7 ± 6.3	34.6 ± 30.5	83.8 ± 41.6	38.9 ± 34.1^∞^	131 ± 29.8	3.3:1	2.5:1
	78 kg (*n* = 106)	51.3 ± 28.5	116.3 ± 43.4^€^	66.4 ± 37.9^*^	163.2 ± 63	3.1 ± 2.2^$^	4.9 ± 3.4	2.8 ± 2.5^&^	5.1 ± 3.3	25.3 ± 24.7^*^	35.8 ± 18.1^&^	48 ± 38.5^*^	130.3 ± 63.1	3.1:1	2.5:1
	>78 kg (*n* = 82)^1^	47.7 ± 25.4	87.9 ± 0	68.3 ± 38.9	159.8 ± 0	2 ± 2.1^Ł^	6.5 ± 0	2.3 ± 2.6	3.9 ± 0	25.3 ± 20.9^μ^	21.2 ± 0	49.4 ± 44.3	78.8 ± 0	2.9:1	3.5:1

RT, regular time; GS, golden score. ^1^At 78 kg division from 2020 cycle, there was only one GS combat, which made statistical analysis unfeasible. Significant difference between 2016 vs. 2020 cycle: ^*^p < 0.001; ^μ^*p* = 0.001; ^β^*p* = 0.003; ^◘^*p* = 0.006; ^Ω^*p* = 0.01; ^∞^*p* = 0.011; ^$^*p* = 0.012; ^ж^*p* = 0.016; ^€^*p* = 0.017; ^Ξ^*p* = 0.019; ^Ł^*p* = 0.02; ^ω^*p* = 0.021; ^α^*p* = 0.023; ^ʮ^*p* = 0.026; ^#^*p* = 0.027; ^&^*p* = 0.031.

In the analysis by weight division of RT combats, there were situations that differed significantly between the Olympic cycles, and 2020 cycle athletes: from 48 kg spent less time in all phases (approach: *t*_(209)_ = 3.841; *p* < 0.001; r = 0.27; gripping: *t*_(209)_ = 2.246; *p* = 0.026; *r* = 0.16; attack: t_(209)_ = 5.138; *p<*0.001; *r =* 0.33; defense: *t*_(209)_ = 3.855; *p* < 0.001; *r* = 0.26; groundwork: *t*_(209)_ = 4.860; *p* < 0.001; *r* = 0.32; pause: *t*_(209)_ = 4.001; *p* < 0.001; *r* = 0.29); from 52 kg spent less time in the gripping (*U =* 1,198; *p* = 0.003; *r* = −0.28) and attack (*t*_(117)_ = 2.379; *p* = 0.019; *r* = 0.21) phases; from 63 kg spent less time in the approach (*t*_(164)_ = 2.619; *p* = 0.01; *r* = 0.2), defense (*U =* 2350.5; *p* = 0.001; *r* = −0.27), groundwork (*t*_(164)_ = 2.801; *p* = 0.006; *r* = 0.16) and pause (*t*_(164)_ = 2.979; *p* = 0.003; *r* = 0.19) phases; from 70 kg spent less time in the gripping (*U =* 973; *p* < 0.001; *r* = −0.38), attack (*t*_(116)_ = 2.317; *p* = 0.023; *r* = 0.21), defense (*t*_(116)_ = 2.839; *p* = 0.006; *r* = 0.25) and pause (*t*_(116)_ = 2.589; *p* = 0.011; *r* = 0.23) phases; from 78 kg spent less time in the gripping (*t*_(182)_ = 4.139; *p<*0.001; *r* = 0.29), attack (t_(182)_ = 2.55; *p* = 0.012; *r =* 0.17), defense (*U =* 3399.5; *p* = 0.031; *r =* −0.16), groundwork (t_(182)_ = 4.157; *p<*0.001; *r =* 0.29), and pause (t_(182)_ = 4.219; *p<*0.001; *r =* 0.29) phases; from ˃78 kg spent less time in the attack (*U =* 2,453; p = 0.02; *r =* −0.19) and groundwork (t_(156)_ = 3.347; p = 0.001; *r =* 0.29) phases (Table 2).

Although the effort/pause ratio varied by weight division in both cycles, the values in 2020 were closer between weight divisions. In 2016, combats that ended in RT had the lowest value in the 63 kg class (2.5:1) and the highest value in the 52 kg class (3.5:1). In the 2020 cycle, the lowest effort/pause ratio was in the ˃ 78 kg division (2.9:1), and the highest ratio was in the 48, 52, and 70 kg divisions (3.3:1; Table 2).

In the analysis by weight division of GS combats, there were situations that differed significantly between the Olympic cycles, and 2020 cycle athletes: from 48 kg spent more time in the gripping (*t*_(52)_ = −3.778; *p<*0.001; *r =* 0.47) and groundwork (t_(52)_ = −2.390; *p* = 0.021; *r* = 0.23) phases; from 52 kg spent more time in the approach (t_(23)_ = −2.774; p = 0.012; *r* = 0.41) phase; from 57 kg spent more time in the groundwork (*U =* 87; *p* = 0.016; *r* = −0.40) phase; from 78 kg spent more time in the approach (t_(26)_ = −2.539; *p* = 0.017; *r =* 0.17) and less time in the groundwork (t_(26)_ = 5.234; p = 0.031; *r =* 0.89) phases. The weight division ˃78 kg only had 1 occurrence of GS in the 2020 cycle and 5 occurrences in the 2016 cycle, which made the statistical analysis between the cycles unfeasible (Table 2).

In addition, in the 2016 cycle, combats that ended in the GS had the lowest value in the 70 and ˃ 78 kg classes (1.8:1) and the highest value in the 52 kg division (3:1). In the 2020 cycle, the lowest effort/pause ratios were in the 48 kg and 63 kg divisions (2.4:1) and the highest ratio was in the ˃ 78 kg division (3.5:1; referring to 1 combat time; Table 2).

## Discussion

This study compared the time of female judo combat phases in international competitions between the 2016 and 2020 Olympic cycles by weight division and by moment at the end of combat. For a more organized discussion of the results, we have divided the findings into two subchapters: (a) time of combat phases; (b) time of combat phases by end moment.

### Time of the combat phases

Our main results showed that, despite the official combat time for female judo being 4 min in both Olympic cycles analyzed, athletes from the 2020 cycle spent less time in the gripping, attack, defense, groundwork, and pause phases than athletes in the 2016 cycle, resulting in a reduction in the offensive phases of combat. Although a slight increase in the effort: pause ratio was observed between Olympic cycles, the analysis by weight division in 2016 showed great diversity in the effort: pause ratio (↓value: 63 kg; ↑value: 52 kg), whereas the effort: pause ratio was similar between weight divisions in the 2020 cycle, with a reduction in the value for some categories (52 and 57 kg) and an increase in the value for the other weight divisions (Table 1). These data show that specific analyses by weight are important for a better understanding of the time demand of combat agents.

Some similarities characterized and differentiated the temporal demands between weight divisions. In both Olympic cycles, the 48 kg athletes had the longest approach, attack, defense, groundwork and pause times, despite they had spent less time in the attack, defense and pause phases in the 2020 Olympic cycle compared to the 2016 (Table 1). Athletes with lower body mass are generally more agile and quick in their movements and need to spend more time in the approach phase to perform an efficient grip and apply immediate attack techniques; consequently, defending against the opponent is difficult (Kashiwagura and Franchini, 2022). Moreover, the approach and groundwork phases can be used to manage combat time and avoid opponent attacks after obtaining a score. In line with our results, analyses of athletes from international competitions in 2011–2012 showed that lighter divisions spent more time in the approach and groundwork phases than heavier divisions. Adam et al. (2013) found that the 2012 Olympic champion of the 48 kg division showed greater versatility in the application of techniques than other weight divisions (attack versatility index: 48 kg = 32; 52 kg = 26; 70 kg = 10; ˃78 kg = 16).

Conversely, the 78 kg athletes had the longest gripping time in both Olympic cycles and they had a time reduction only in the groundwork phase between Olympic cycles (Table 1). Athletes with a higher body mass strategically spend more time maintaining the grip and positioning their body in the best way to carry out the attack (Courel et al., 2014) because the risk of losing the combat during groundwork is high if the attack is not successful, mainly due to immobilization. In fact, Adam et al. (2013) observed that the groundwork attack efficiency indices were highest for the 63 kg and 78 kg divisions of the 2012 women’s Olympic champions (efficiency index: 63 kg = 6 and 78 kg = 5 vs. 48 kg, 52 kg, 57 kg, ˃ 78 kg = 0 and 70 kg = 1.3). In this sense, greater efficiency of attacks in standing combat could explain the reduction in groundwork time in the 2020 cycle, which also occurred for the ˃ 78 kg athletes.

The ˃78 kg athletes had the shortest approach, attack, defense, and groundwork times in both Olympic Cycles. In addition, they spent less time in the gripping, attack and groundwork phases in the 2020 Olympic cycle compared to the 2016 (Table 1). These data demonstrate that the heaviest female weight division spends little time being able to hold the *judogi* and performing offensive actions, irrespective of the rule in force in the Olympic cycle. The reason for this behavior is twofold: athletes either minimize the physical wear and tear caused by the dispute of grips and attack actions, which can be greater because of the movement speed/body mass ratio (Courel et al., 2014; Barreto et al., 2019), or the ˃78 kg attack actions are highly effective. In fact, Ceylan and Balci (2021) analyzed combats from 2018 to 2019 and observed that 5 of the 6 combats in the ˃ 78 kg ended with *Ippon* before the end of the regular time. Thus, the data suggest that the 48, 78, and ˃ 78 kg weight divisions have specific profiles and use different combat strategies.

### Time of the combat phases by end moment

When analyzing the total time of the combat phases by weight divisions between Olympic cycles, many temporal similarities persisted between the cycles, as demonstrated in the previous subchapter. However, when we analyzed the combats by the moment they ended (RT vs. GS), we identified with greater clarity the effects that the rule changes had on the female combat time, as there was a reduction in time in all phases of RT combats, an increase in the approach and maintenance of time spent in the other phases of the GS combats (Table 2).

Analyzing by weight division and end-of-combat, we observed that in the 2020 cycle, the 48 kg reduced significantly the time in all phases in the RT combats (Table 2). These results can be explained by the rule changes that occurred between the Olympic cycles. Unlike in the 2016 cycle, as of the 2017 rule change, penalties no longer decide the winner of combats that continue until RT in the event of a tie (Federation, 2013, 2017); therefore, the best strategy in the 2020 cycle was to win as quickly as possible to avoid the GS. Therefore, while in the 2016 cycle the 48 kg spent the most time in the approach phase (both RT and GS), in the 2020 cycle, this time phase was close to the approach time spent by the 52, 57, and 63 kg weight divisions (both RT and GS).

Athletes unable to perform efficient attacks in RT required a GS. The 48 kg increased the GS gripping time in the 2020 cycle (Table 2). These data suggest that 48 kg athletes from 2020 cycle, who were unable to win the combat in regular time, performed defensive actions in the Golden Score, as they spent a lot of time holding the *judogi*, without performing attacks. It is believed that these athletes win the combat on the Golden Score by penalty. Ceylan et al. (2022), who analyzed 5,111 judo combats from 2018 and 2019 (women = 2,191; men = 2,920), identified that the possibility of a GS was greater in light and middle weight divisions and that the number of penalties increased this possibility.

In the 2020 cycle, almost all weight divisions reduced the time spent in the RT attack phase (except 57 kg and 63 kg), the 52 kg and 78 kg increased the time spent in the GS approach phase, and the GS period for the attack and defense phases did not change between Olympic cycles for any weight division (Table 2). These results indicate that the athletes who could not win the combat by the RT insisted on the strategy of seeking the opponent’s disqualification to win. With the 2018 rule change, only the accumulation of 3 opponents’ *Shido* (*Hansokumake*) or the 1st score would result in a victory in GS (International Judo Federation, 2017b), unlike in the 2016 cycle, when the 1st opponent’s *Shido* determined the winner (International Judo Federation, 2013).

A limitation of our study is that we did not control the type of decision (combats ending by scoring or disqualification). However, regarding the penalties committed in the 2020 cycle, Balci and Ceylan (2020) analyzed the 2018–2019 senior world judo championships and found that the most committed prohibited actions in combat were non-combativity (common for those who held the grip without making attacks) and avoid grip (common for those who spent a lot of time in the approach phase). Kajmovic et al. (2022) carried out a study that analyzed 2041 penalties committed by female judoka from competitions between 2017 and 2021. They identified that the main penalties were non-combativity (41.6%), avoid grip (16.2%) and false attack (15%). Therefore, it seems that the rule change between cycles did not boost the performance of offensive actions.

Conversely, the groundwork phase may have become relevant to define the GS winner for 48 and 57 kg athletes from 2020 cycle, as these athletes increased the GS groundwork time (Table 2). However, this combat phase could be used either to win combat in a continuous action, or to avoid penalties after an unsuccessful attack. On the other hand, the 78 kg athletes modified the use of the GS groundwork phase between Olympic cycles. While in the 2016 cycle, the 78 kg were the ones who spent the most time in the GS groundwork phase, in the 2020 cycle they spent the shortest time in this phase (Table 2).

In our data collection we did not count the effort cycles, because the initial objective was just to observe if there was a change in the time spent in the combat phases with different rules. To alleviate this limitation, we calculated the effort: pause ratio for each phase (Tables 1 and 2). The effort: pause ratio that we observed was consistent with data from other studies that showed that an average of 11 cycles of effort occur in judo combat, ranging from 20 to 30 s of effort and 10 s of rest (Marcon et al., 2010; Franchini et al., 2013). However, in our data, the 48, 63, 70, and 78 kg divisions reduced the pause time and increased the effort: pause ratio of RT combats in the 2020 cycle compared to the 2016 cycle (Table 2), which suggests fewer combat interruptions in the 2020 cycle and that these athletes won combats in the RT by score. On the other hand, there was no significant difference in the GS pause phase between Olympic cycles.

The analysis of judo combats allows the collection of a large number of variables, and therefore, researchers need to choose which variables will be analyzed to contemplate the writing of an article. Our data showed some changes in the temporal behaviors between cycles for some weight divisions. These results highlight the importance of understanding what happens in each weight division and analyzing the moment of the end of the combat to prepare more specific training sessions according to the athlete’s profile, i.e., if the athletes usually finish the combat until RT or combat ends in the GS. However, another limitation of this study is that only the temporal analysis of the combat was performed, that is, data on the actions performed by the athletes in each phase were not collected. It could influence the training planning decisions by judo coaches. Therefore, we suggest that other studies identify the actions (type of approach, grip and techniques) performed by athletes in each Olympic cycle to highlight possible difference in the technical actions performed.

## Practical application

We created a table that summarizes the main temporal changes by Olympic cycle found in this study (Table 3) to allow judo coaches to understand and apply the results of this study in practice. Specifically, 2020 athletes spent less time in all combat phases compared to the 2016 cycle for combat that continued until RT, indicating that athletes were able to win combat faster than they did before. In the combats that ended in the GS, the 2020 athletes spent more time in the approach phase than in the 2016 cycle, which suggests that athletes spent time on non-offensive actions and searched for the opposing penalty at the expense of *Ippon*.

**Table 3:** Significant changes in female judo combat phases in the 2020 Olympic cycle compared to the 2016 cycle (*p* ≤ 0.05).

Weight division	Approach	Gripping	Attack	Defense	Groundwork	Pause
All weight divisions	--	↓	↓	↓	↓	↓
48 kg	--	--	↓	↓	--	↓
52 kg	--	↓	--	--	--	--
57 kg	--	--	--	--	↑	--
63 kg	--	--	--	↓	--	--
70 kg	--	↓	↓	--	--	--
78 kg	--	--	--	--	↓	--
˃78 kg	--	↓	↓	--	↓	--
Weight division	Combats ended until the Regular Time					
	Approach	Gripping	Attack	Defense	Groundwork	Pause
All weight divisions	↓	↓	↓	↓	↓	↓
48 kg	↓	↓	↓	↓	↓	↓
52 kg	--	↓	↓	--	--	--
57 kg	--	--	--	--	--	--
63 kg	↓	--	--	↓	↓	↓
70 kg	--	↓	↓	↓	--	↓
78 kg	--	↓	↓	↓	↓	↓
˃78 kg	--	--	↓	--	↓	--
Weight division	Combats ended in the *Golden Score*					
	Approach	Gripping	Attack	Defense	Groundwork	Pause
All weight divisions	↑	--	--	--	--	--
48 kg	--	↑	--	--	↑	--
52 kg	↑	--	--	--	--	--
57 kg	--	--	--	--	↑	--
63 kg	--	--	--	--	--	--
70 kg	--	--	--	--	--	--
78 kg	↑	--	--	--	↓	--
˃78 kg^*^						

-- kept the average combat time; ↑ increased the average combat time; ↓ decreased the average combat time. ^*^ Occurrence of only 1 combat made statistical analysis unfeasible.

When the analysis was stratified by category and end-of-combat, a comparison between the 2020 and 2016 cycles showed the following: (a) the 48 kg reduced the time spent in all phases of combats finished until RT; they increased the gripping time and reduced the groundwork time in the GS combats; (b) The 63, 70, and 78 kg divisions reduced the time spent in almost every phase of RT combat (except for 63 kg = gripping and attack; 70 kg = approach and groundwork; 78 kg = approach). (c) The 78 kg division increased the approach time and reduced the groundwork time in the combats ended in the GS. Thus, our main results showed that performing specific analyses by weight division and separating athletes who usually finish combat in RT from those who usually require GS are important considerations to understand the characteristics of each group.

## Conclusion

In general, we found that the athletes from the 2020 cycle reduced the time spent on offensive actions (attack, defense and groundwork) compared to athletes from the 2016 cycle. In addition, the weight divisions in the 2016 cycle presented greater diversity in the values of the effort: pause ratio, whereas these values were similar for athletes of the 2020 cycle. However, we were only able to specifically detect how these changes occurred when we analyzed the combats by weight division and end-of-combat time. In summary, the temporal behavior of the combat changed between the Olympic cycles as new rules were implemented.

## Data availability statement

The raw data supporting the conclusions of this article will be made available by the authors, without undue reservation.

## Author contributions

LBMB, BM, and CJB participated in the research concept, study design, and literature review. LB participated in the data collection. LBMB, RJSS, EAA-M, NLB, NMS, DASS, HZ, BM and CJB participated in the data analysis and interpretation, statistical analyses and writing of the manuscript. All authors contributed to the article and approved the submitted version.

## Funding

This study was financed in part by the Coordenação de Aperfeiçoamento de Pessoal de Nível Superior - Brasil (CAPES)—Finance Code 001. LB received a PDSE/CAPES Scholarship. Grant# 88881.622965/2021-1.

## Conflict of interest

The authors declare that the research was conducted in the absence of any commercial or financial relationships that could be construed as a potential conflict of interest.

## Publisher’s note

All claims expressed in this article are solely those of the authors and do not necessarily represent those of their affiliated organizations, or those of the publisher, the editors and the reviewers. Any product that may be evaluated in this article, or claim that may be made by its manufacturer, is not guaranteed or endorsed by the publisher.
